# Estimating and reducing dose received by cardiac devices for patients undergoing radiotherapy

**DOI:** 10.1120/jacmp.v16i6.5317

**Published:** 2015-11-08

**Authors:** Alexandra Bourgouin, Nicolas Varfalvy, Louis Archambault

**Affiliations:** ^1^ Département de physique de génie physique et d'optique, Université Laval Québec QC Canada; ^2^ Département de radio‐oncologie Centre Hospitalier Universitaire de Québec Québec QC Canada

**Keywords:** CIED, shielding, plastic scintillation detector, treatment planning system, out‐of‐field dose

## Abstract

The objectives of this project are to quantify the dose reduction effect provided by a lead shield for patients with cardiac implantable electronic devices (CIED) during a clinically realistic radiation treatment on phantom and to provide a simple model of dose estimation to predict dose received by CIED in a wide range of situations. The shield used in this project is composed of a lead sheet wrapped in thermoplastic. Dose measurements were made with a plastic scintillation detector (PSD). The phantom was treated with ten different plans. Three of these cases were treated with intensity‐modulated radiation therapy (IMRT) and the others received standard 3D conformal radiation therapy (3D CRT). Lateral dose measurement for photon fields was made to establish a dose prediction model. On average, the use of the lead shield reduced the dose to CIEDs by 19%±13%. Dose reduction was most important for breast cases, with a mean reduction of 31%±15%. In three cases, the total dose reduction was more than 25 cGy over the complete treatment. For the three IMRT cases, the mean dose reduction was 11%±9%. On average, the difference between the TPS prediction and the measurement was 71%, while it was only 14% for the dose prediction model. It was demonstrated that a lead shield can be efficiently used for reducing doses to CIED with a wide range of clinical plans. In patients treated with IMRT modality treatment, the shielding should be used only for those with more than two anterior fields over seven fields. In the case of 3D CRT patients, the shielding should be used for those with a dose on the CIED higher than 50 cGy and with a reduction of dose higher than 10 cGy. The dose prediction model developed in this study can be an easy way to have a better estimation of the out‐of‐field dose than the TPS.

PACS number(s): 87.55.N, 87.55.km

## I. INTRODUCTION

Because of the aging population, the number of cardiac implantable electronic devices (CIED) encountered in radiation therapy is increasing. It had been shown that the CIEDs may be damaged by radiation.^(1)^ There are two major types of CIED: pacemaker and implantable cardioverter‐defibrillator (ICD). The pacemaker and ICD have the same basic function, but ICDs are also capable of defibrillation. In 2002, a large study by Mouton et al.^(1)^ was conducted on 96 pacemakers irradiated by high‐energy photon beams. Permanent silence was detected at 0.5 Gy and a compatible slowed‐down rate, a failure that can induce discomfort, was also detected at 0.05 Gy. The proportion of important failures under a cumulated prescription dose of 2 Gy was 6%. Mouton et al.^(1)^ showed that there is no threshold dose to the pacemakers below which the risk of failure is absent.

According to practice guidelines set by AAPM Task Group 34,^(2)^ CIEDs must not be present in the treatment beams.^3^, ^4^ Moreover, the CIED's cumulated dose should either be estimated or measured during treatment. However, it is difficult to estimate precisely the dose to CIED. It is well known that out‐of‐field doses beyond the beam penumbra are not well modeled by TPS.^(5)^ Although dose received by cardiac devices should remain below 2 Gy,^(6)^ CIEDs failures are not predictable and might happen before this threshold.^(1)^ Hurkmans et al.^(7)^ published guidelines for the management of patients with pacemakers or ICDs in radiation oncology. In that article, they suggested categorizing the patient risk according to the dependency of the patient on her or his CIED and the estimated dose to the CIED. Furthermore, the estimation of the dose to the CIED is based only on the zone of the patient's tumor. Three categories of dose level are suggested: more than 10 Gy for a tumor in the upper zone of the thorax and the neck, between 2 and 10 Gy for one in the medium zone of the thorax and in the lower‐half section of the head, and below 2 Gy for a tumor in the rest of the body.

To reduce the dose received by a CIED during radiation therapy treatments, some clinics use a lead sheet (wrapped in thermoplastic) placed directly on the patient's skin above the sensitive device. The objectives of this study are: 1) to retrospectively verify and quantify the impact of using a shield to reduce dose to CIEDs for ten different radiotherapy treatment plans (RTP), and 2) to build a dose prediction model for a better estimation of out‐of‐field doses based on phantom measurements.

## II. MATERIALS AND METHODS

Measurements in this study were performed on a Varian Clinac iX (Varian Medical Systems, Palo Alto, CA). Ten previously treated clinical plans were used. In each case, fields from the original plans were copied onto the CT scan of the phantom and doses were computed by the TPS. For each case, the phantom was placed as close as possible to the patient positioning during the treatment.

### A. Dosimeter

For this study, the dosimeter selected is a plastic scintillation detector (PSD) because of its dosimetric advantages for out‐of‐field dose measurements: energy independence, reproducibility, angular independence, water equivalence, dose and dose rate linearity^8^, ^9^, ^10^, ^11^, ^12^ The detector used in this project is the first commercial miniature PSD: the Exradin W1 (Standard Imaging Inc., Middleton, WI). The scintillator dimensions are 1 mm in diameter by 3 mm long ($2.355\;{\text{mm}}^{3}$). The light is transported over a clear optical fiber to a photodiode. The output is read by a double‐channel electrometer, the SuperMAX Electrometer (Standard Imaging Inc.). The calibration of Exradin W1 was performed following the manufacturer's method. The Exradin W1 was positioned on the phantom to measure the dose at a position equivalent to the location of the CIED.

### B. The lead shield

The lead shield is a thin sheet of lead wrapped in thermoplastic. The lead sheet has a thickness of 1.6 mm and an area of $10\times 10{\text{cm}}^{2}$. The thickness of the thermoplastic is 2 mm above, 4 mm under, and 2 mm on the sides of the shield. The shield used in this project is the same as the one used during the treatment of patients. The shielding is sufficiently flexible to adapt to the shape of the patient's collarbone.

### C. Treatment planning system

The treatment planning system used in this study was Pinnacle^3^ version 9.6 (Philips Healthcare, Andover, MA). The phantom was scanned by a CT and exported to the TPS. Then, the fields from the original plan were copied onto the phantom dataset (see next section for details). Dose calculation by the TPS was used to evaluate the dose at the location of the CIED and to measure the distance between the 50% isodose line and the CIED ($D_{50\text{IL}}$). The $D_{50\text{IL}}$ was determined using the isodose line 50% of the whole treatment. The position of the W1 in the phantom was converted to a sphere of 2 mm radius ($V_{W1}$). The volume defined by the isodose line 50% ($V_{50\text{IL}}$) was expanded until it reached VW1. $D_{50\text{IL}}$ was then simply equal to the radius of the expansion.

### D. The phantom

**Figure 1:**
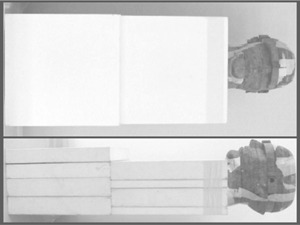
Picture of the torso phantom made of solid water with an anthropomorphic head used in this work.

The phantom is shown on Fig 1. The head of the phantom was made of a water‐equivalent medium. The body of the phantom was made of solid water and bolus material. Bolus material was used above the W1 to minimize the presence of air gaps surrounding the detector, which could induce an error smaller than 1%. The upper section of the body measured $30\times 30\times 16.5\;{\text{cm}}^{3}$ (solid water plus bolus) and the lower section measured $30\times 30\times 20\;{\text{cm}}^{3}$ (solid water only). The phantom's isocenter was positioned to reproduce the treatment isocenter of the patient. For each case, the distance between the patient's isocenter and the middle of the patient's torso was measured on the CT images of the patient. The same distance was then used to set up the phantom. This process was repeated laterally by measuring the distance between shoulders and isocenter. In a similar manner, the detector was positioned in the phantom at a position equivalent to the center of the patient's CIED based on distances measured on the original CT scan. The difference between depths of a patient's CIED and isocenter was measured in the TPS. The same difference of depth was used for the depth of the scintillator in phantom to reproduce the depth of the CIED in patient. When necessary, clinical accessories were placed between the couch and the phantom to increase distance and thus avoid interception of the treatment couch by the radiation beam.

### E. Dose prediction model from lateral‐dose measurements

Dose was measured on the lateral axis (i.e., the left‐right axis of the patient) using the same water phantom as in the previous section. The goal of these measurements was to establish a simple dose prediction model. In this simple model, the depth of the CIED is not considered. This choice was made to keep the model as simple as possible. This is justified by the fact that the out‐of‐field dose is relatively stable at shallow depths.^13^, ^14^ All measurements were made at a depth of 1.5 cm, which is the averaged CIED depth seen in patients. Both 6 MV and 23 MV photon beams were studied. The source‐to‐surface distance was 95.5 cm for anterior fields and 88 cm for posterior fields. Measurements were made for field sizes of 5×5 and $10\times 10{\text{cm}}^{2}$. The dose delivered was 100 MU. The W1 was at 5 cm from the left and top edges of the phantom. The isocenter was at 5 cm from the top edge of the phantom and at a depth of 4.5 cm. The positions of the isocenter, in the lateral axis, changed depending on the out‐of‐field distance to achieve. Five measurement positions were used: 2, 3, 5, 10, and 15 cm out‐of‐field. All measurements were made with and without lead shielding for anterior and posterior fields.

Since the dose decreases exponentially^15^, ^16^, ^17^ with the out‐of‐field distance, the basis of our model is an exponential function. In regard to the conclusion of Zang et al.,^(18)^ a simple exponential decay function has been selected rather than a double exponential function, shown in Eq. (1).

(1) $$D=a^{•}e^{-b}+c$$

The multiplying factor, *a*, and the exponential factor, *‐b*, depend on the energy, the size of the field, and its incidence (AP or PA).

### F. Selection of cases and measurements of each field

The cases selected observed two selection criteria: 1) patients carrying CIED during treatment in external beam radiotherapy, and 2) $D_{50\text{IL}}$ less than 15 cm. Information on treatment region, modality, energies, dose prescription and position of the detector in the phantom is presented in Table 1. The measurement of dose for each field was repeated six times. The analysis is made considering the whole treatment and not just one fraction. All IMRT fields were treated as step‐and‐shoot. RTP 2 used two dose levels, one at 56 Gy and the second at 68 Gy. RTP 4 was a palliative treatment for both the H&N region (vertebra C2‐C3) and for the lung (bronchial compression); four square fields without MLC were used for this treatment and two were lateral fields (gantry at 90° or 270°) for the vertebra. RTP 4 was a palliative case. Breast treatments used tangential fields for the gland and oblique fields for the nodes. RTP 9 were treated for axillary nodes (2 Gy/Fraction, 23 fractions, 2 oblique fields at 23 MV) and supraclavicular (2 Gy/Fraction, 20 fractions, 1 anterior oblique field at 23 MV) in addition to treatment for right breast with four tangential field (2.25 Gy/Fraction, 20 fractions, 2 fields at 6 MV and 2 fields at 23 MV). For RTP 10, the supraclavicular nodes were treated to 46 Gy and the left breast was treated to 45 Gy.

**Table 1:** Treatment information of RTPs, details about out‐of‐field distance and position of W1 in phantom

*RTP*	*1*	2	*3*	*4*	*5*	*6*	7	*8*	*9*	*10*
Treatment region	H&N	H&N, 2 VC	Lung	H&N and lung	Lung	Lung	Lung	Left breast	Right breast / nodes	Left breast / nodes
Treatment modality	IMRT	IMRT	IMRT	3D CRT	3D CRT	3D CRT	3D CRT	3D CRT	3D CRT	3D CRT
Dose prescribed (Gy)	56	56 / 68	45	40	16	30	50	43	Breast; 45 Nodes; (86)	Breast; 45 Nodes (46)
Number of 6 MV beams	7	7	6	2	0	0	2	2	2	2
Number of 23 MV beams	0	0	0	2	3	2	1	2	5	3
Lateral distance between W1 and isocenter (cm)	9.9	10.2	11.6	13.2	12.7	12.1	5.3	1.6	12.3	15.0
Longitudinal distance between W1 and isocenter (cm)	13.2	14.5	9.7	6.4	12.7	6.2	6.4	1.5	4.8	1.5
Depth of W1 (cm)	1.5	1.5	1.0	1.5	1.0	2.5	1.5	1.5	1.0	1.0
$D_{50\text{IL}}$ (cm)	7.0	4.1	7.6	7.8	12.6	8.5	2.4	4.8	6.3	8.7

## III. RESULTS & DISCUSSION

### A. Dose prediction model

**Figure 2:**
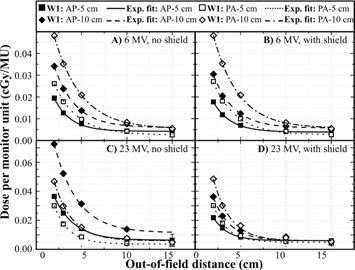
Measurement of the dose (cGy) by MU delivered by the linac for a 6 and 23 MV beam as a function of the out‐of‐field distance: (a) and (b) anterior and posterior fields of 5 and 10 cm side at 6 MV; (c) and (d) anterior and posterior fields of 5 and 10 cm side at 23 MV.

The results of the lateral dose measurements at 6 MV and 23 MV are shown in Fig. 2 with the exponential decay function. The standard deviation (SD) is between 0.55 and 0.01 cGy and the mean SD is 0.06 cGy. The uncertainties are estimated to be less than 7%, which is smaller than the symbols on Fig. 2. All regression coefficients ($R^{2}$) are higher than 0.99, except for the anterior $10\times 10{\text{cm}}^{2}$ field at 23 MV with shield, $R^{2}=0.98$.

The results for lateral dose measurement show that for a constant out‐of‐field distance and beam size for anterior fields, the dose is higher for the 23 MV beam compared to 6 MV beam. This observation is in agreement with other publications.^14^, ^16^ It was also observed that the out‐of‐field dose, for the same irradiation conditions, are higher for the larger beam, which is also in agreement with the literature.^13^, ^14^, ^19^, ^20^, ^21^ The out‐of‐field doses were also higher for posterior fields than for anterior fields for a 6 MV beam. For a PDD curve 5 cm out‐of‐field for a 6 MV beam, the dose is higher at 15 cm depth than at 1.5 cm and the difference in the dose at these two depths is higher for a larger field.^16^, ^21^ These results are in agreement with our measurements. For the 23 MV beam, the opposite is observed; the dose is higher for anterior fields, which is also in agreement with measurements by Vlachopoulou et al.^(16)^

The reduction of dose by shielding is more important for the higher energy. This result is in agreement with the literature and the fact that the lead sheet is shielding in major part the electron contamination and the radiation leakage from the head of the linac. The electron contamination and the leakage increase with the energy of the beam.^(13)^

In accordance with these observations, fields are separated into different groups as shown in Table 2. They are categorized based on beam energy, the presence (or not) of shielding, and the beam incidence (AP or PA). The anterior fields regroup all beams with a gantry angle between 0° and 90° or 270° and 360° and posterior fields regroup all beams with a gantry angle between 90° and 270°. Fields are also separated in groups based on the volume of the 50% isodose line, which can be easily obtained from the TPS. Lateral dose measurements show that a categorization based on field size was necessary. In our model, the volume of the 50% isodose line plays this role. It was found that using a threshold of 1,500 cm^3^ gave optimal results. On average, the difference between the estimated doses is 5% closer to the dose measured by choosing a threshold at $1,500\;{\text{cm}}^{3}$ rather than 1,000 or $2,000\;{\text{cm}}^{3}$.

**Table 2:** Factors α, β, and γ of Eq. (2), according to beam parameter

*Energy Beam*	*Type Of Field*	*Volume of the Isodose 50%*	*α*	*β*	*γ*
6 MV no shield	Anterior	≤1500	0.0448	1.83	0.00422
		≥1500	0.0661	2.33	0.00591
	Posterior	≤1500	0.0516	2.53	0.00256
		≥1500	0.0829	2.97	0.00534
23 MV no shield	Anterior	≤1500	0.0733	2.24	0.00623
		≥1500	0.133	2.55	0.0115
	Posterior	≤1500	0.0892	1.64	0.00363
		≥1500	0.112	1.99	0.00589
6 MV shielded	Anterior	≤1500	0.0383	1.94	0.00386
		≥1500	0.0587	2.28	0.00572
	Posterior	≤1500	0.0534	2.49	0.00269
		≥1500	0.0869	2.79	0.00564
23 MV shielded	Anterior	≤1500	0.0541	1.63	0.00593
		≥1500	0.0879	1.85	0.00594
	Posterior	≤1500	0.0910	1.56	0.00484
		≥1500	0.122	1.89	0.00586

The dose prediction model can only be used for photons 3D CRT fields in distances between 2 and 15 cm from the primary beam. The final model is based on an exponential equation as shown below:

(2) $$D=N_{MU}(\alpha^{•}e^{-d/\beta +\gamma })$$

where *D* is the out‐of‐field dose, $N_{MU}$ is the number of monitor units prescribed for the field, and *d* is the out‐of‐field distance in cm. Factors α, β, and γ depend on the beam parameters. The units of α and γ are cGy/MU and β is in cm. The values of the three parameters of Eq. (2) are determined by comparing the beam parameters to those of Table 2. This model is used in the following sections to predict CIED doses.

### B. Dose measurement on phantom

**Figure 3:**
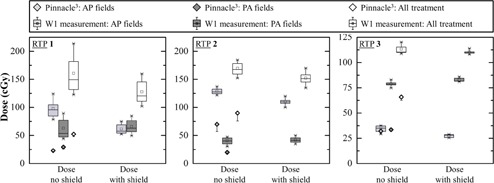
Dose measurement by W1 and TPS calculation for anterior fields, posterior fields, and for the complete treatment for all IMRT modality plans. Anterior and posterior results are the total dose measured or calculated for all anterior or posterior fields of the RTP. Measurements of W1 are presented in box plot and TPS calculation results are presented with its minus dose deviation.

**Figure 4:**
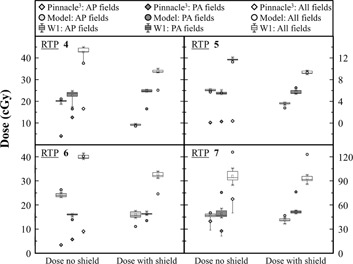
Dose measurement by W1 and TPS calculation for anterior fields, posterior fields, and for the complete treatment for RTPs with a 3D CRT modality in the head‐and‐neck and lung regions. Anterior and posterior results are the total dose measured or calculated of all anterior or posterior fields of the treatment. Measurements of W1 are presented in box plot and TPS calculation results are presented with its minus dose deviation.

**Figure 5:**
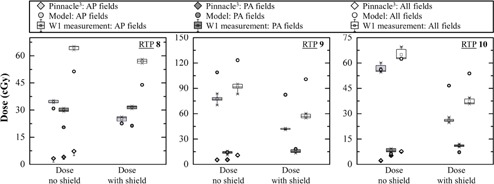
Dose measurement by W1 and TPS calculation for anterior fields, posterior fields, and for the complete treatment for RTPs with a 3D CRT modality for a breast cancer. Anterior and posterior results are the total dose measured or calculated of all anterior or posterior fields of the RTP. Measurements of W1 are presented in box plot and TPS calculation results are presented with its minus dose deviation.

Measurements for all clinical plans are shown in Figs. 3, 4, and 5, and in Table 3. TPS doses are obtained by averaging the dose over a sphere of 5 mm radius. The sphere is located at the same position as the W1 on the phantom. For better clarity in the Figure, only half‐error bars are shown. The box plots are obtained from six repeated measurements.

**Table 3:** Doses measured, modeled, and calculated by TPS

	*RTP*		*1*	*2*	*3*	*4*	*5*	*6*	7	*8*	*9*	*10*
Reduction of Doses Absolute (cGy) and Relative (%) Value	Modeled	Value cGy	No applied	No applied	No applied	A: −12	A: −2	A: −15.6	A: −2.8	A: −7.4	A: −23	A: −9
						R: −33	R: −18	R: −39	R: −2.3	R: −14	R: −18	R: −14
	Measured	Value cGy	A: −33±12	A: −17±3	A: −3±6	A: −9±3	A: −2.4±0.3	A: −7.6±0.8	A: −3.4±3	A: −7.5±0.5	A: −34±6	A: −28±3
			R: −21±12	R: −10±3	R: −3±5	R: −21±7	R: −20±3	R: −19±2	R: −3.6±3.9	R: −12±1	R: −37±8	R: −43±7
Shielded Doses (cGy)	Modeled	Diff. with measured %	No applied	No applied	No applied	26	0.3	24	33	23	76	44
		Value cGy	No applied	No applied	No applied	25	9	25	123	44	101	54
	Measured	Value cGy	128±22	152±12	110±2	33.9±0.7	9.3±0.3	32±1	92±4	57±1	57±2	37±2
Unshielded Doses (cGy)	TPS	Diff. with measured %	67	47	42	61	97	77	30	89	88	88
		Value cGy	53	90	66	17	0.4	9	67	7	11	8
	Modeled	Diff. with measured %	No applied	No applied	No applied	12	4	0.3	31	20	35	4
		Value cGy	No applied	No applied	No applied	38	11	40.2	126	51	123	62
	Measured	Value cGy	161±34	170±12	113±4	43±3	11.7±0.3	40.1±0.9	96±8	65±1	91±4	65±3

#### B.1 IMRT cases

Because of the large differences and the complexity of the three IMRT cases, the ratio of the measured out‐of‐field dose and the number of monitor units have been calculated in order to compare dose received by CIED. The ratio of dose / MU for all fields is $[12\pm 1]10^{-3}$ for RTP 1, $[9.6\pm 0.3]10^{-3}$ for RTP 2, and $[17.0\pm 0.6]10^{-3}$ for RTP 3. Higher ratio Dose / MU for RTP 3 is explained by the treatment region. RTP 1 and 2 are in the head‐and‐neck region and RTP 3 in the lung. Differences in the region treated affect the amount of scatter material in the path of the beam. However, only one lung case was measured and two in the head‐and‐neck region, which is not sufficient to draw a clear conclusion.

For IMRT patients, shielding should be recommended for patients with at least two out of seven fields (case of patient 3) in the anterior region. For each IMRT patient, the dose increase from the posterior field is compensated by the reduction of dose for the anterior fields. If possible, it is better to keep the shield in place for the whole treatment to avoid unnecessary reentry into the treatment vault by the therapists.

#### B.2 3D CRT modality cases

For all RTPs with 3D CRT modality in the head‐and‐neck and/or lung regions, the use of shielding is not justified because of the low dose reduction. The increase of doses from posterior fields is compensated by the reduction of dose for the anterior fields, even if the treatment plan counts one anterior field out of three posterior fields.

For RTPs in the breast region, the overall normalized reduction of dose provided by the shielding is on average the highest over all other RTPs. The important weight of 23 MV anterior fields is a factor which influences the total dose reduction and explains why breast cases reach the higher reduction of doses in mean.

The normalized reduction of dose for all RTPs with 3D CRT modality is influenced by: the average out‐of‐field distance of the CIED, the relative weight of the anterior fields, the beam energy, the average size of the fields, and other factors. It was also observed that the reduction of dose is more important for a field energy of 23 MV compared to 6 MV. The larger the dose contribution from anterior beams compared to the dose contribution from posterior fields, the more effective the shielding will be.

#### B.3 All RTPs comparisons

Dose reduction was most important for breast cases with a mean reduction of 31% and a maximum reduction of 43%. The smallest reduction of dose was for lung cases, 2.4 cGy. In three cases, the total dose reduction was more than 25 cGy over the complete treatment. In two cases, the lead shield induced no reduction of dose (−3±6cGy and −3.4±3cGy) over the treatment. For these two cases, one had four posterior fields out of seven fields and the other got anterior fields less energetic than posterior fields. The lead shield reduces doses from anterior fields and slightly increases the dose from posterior fields. For breast cancer, the reduction of dose for 23 MV tangential anterior fields are on average eight times more important than the reduction measured for the same fields but at 6 MV. For three IMRT cases, the mean dose reduction was 11%, while the mean reduction of the four 3D CRT of head and neck and lung cases was 16%. The dose reduction is in the same range for IMRT than 3D CRT. However, the absolute dose received by CIED in IMRT cases is greater (dose reduction of 18 cGy compared with 6 cGy for the 3D CRT cases). The mean normalized reduction of dose was 19% for all measured treatments and the mean absolute reduction of dose was 15 cGy.

### C. Calculation of out‐of‐field dose with the dose prediction model

Over all 3D CRT cases (28 fields), the estimation of the dose by the model is better than TPS in 85% of treatment plans. For all seven 3D CRT RTPs, the calculation of the total dose over the treatment is always better from the model than the TPS calculation. For all fields, the mean error on dose estimation from TPS is 76% and from the dose prediction mode is 14%. On average, the error on dose calculation is 75% smaller for the model compared to TPS calculation. The model can also estimate the dose to the CIED after being shielded, which TPS cannot properly calculate (TPS results give an increase of dose by shielding).

Another purpose of the dose prediction model is to provide a tool to help physicists decide whether or not to use shielding for patients carrying CIEDs. Based on our experience, we established that, in our clinic, shielding should be recommended for 3D CRT patients who would receive ≥50 cGy to their CIED, and for which shielding could provide a dose reduction of ≥10 cGy. Based on these criteria, the decision to use a shield for a given patient will vary, depending on the method used to estimate CIED doses. Considering the measurements as the reference, the TPS improperly calculated the dose in three out of seven cases, which would result in an incorrect shielding decision. The model, however, yielded more accurate information than the TPS and allowed for the proper strategy (shielding or not) to be implemented in all but one RTP. To quantify the statistical performance of the dose prediction model and the TPS, we calculated the sensitivity and the specificity. In this project, sensitivity is defined as the capacity to identify patients that should be shielded. The sensitivity is defined by the following equation:

(3) $$Sensitivity=\frac{N_{TP}}{N_{TP}+N_{FN}}$$

where *N_TP_* is the number of true positives, and $N_{FN}$ is the number of false negatives.

The specificity is defined as the capacity to identify patients that do not need to be shielded. The specificity is defined by the following equation:

(4) $$Specificity==\frac{N_{TN}}{N_{TN}+N_{FP}}$$

where $N_{TN}$ is the number of true negatives, and $N_{FP}$ is the number of false positives.

The sensitivity and the specificity of the dose prediction model are 50% and 100%. The sensitivity is low because there is one true positive and one false negative. For TPS, the sensitivity and the specificity are 0% and 80%, no true positives.

## IV. CONCLUSIONS

It was demonstrated that a lead shield can be efficiently used for reducing doses to CIED for patients treated in radiation therapy. Lead shielding provided a mean dose reduction of 23% over different treatment regions. When the CIED is at less than 15 cm from the treatment fields, placing a lead shield as a skin block is a useful and simple method for achieving dose reduction to radiation‐sensitive cardiac devices. However, the shielding should not be used for any patients having a CIED at less than 15 cm from the isodose line at 50% of the radiation treatment. Shielding should be recommended for 3D CRT patients who would receive a higher dose than 50 cGy to their CIED, and for which shielding could provide a dose reduction larger than 10 cGy. For IMRT patients, shielding should be recommended for patients with a ratio of anterior fields to all fields higher than 2 out of 7. According to this study, for 40% of patients carrying a CIED at less than 15 cm from the 50% isodose line, shielding should be used to reduce the dose to the CIED.

The best way to estimate the dose and the reduction of dose reach by shielding is by making measurements. However, it is not realistic to make this kind of measurement for each patient with a CIED at less than 15 cm out‐of‐field. Another way to get a better estimation of the dose than TPS calculation is to use the dose prediction model developed in this study. The estimation of the dose by this model is, on average, 14% from the dose measurement without shielding and 33% from the measurement with shielding. The dose prediction model can be applied for patients having CIEDs at different depths and at an out‐of‐field distance smaller than 15 cm. This dose prediction model can be used to estimate the CIED dose from a treatment having multiple oblique beams with both photon energy beams 6 and 23 MV.

## ACKNOWLEDGMENTS

This research is supported in part by Natural Sciences and Engineering Research Council of Canada (NSERC) Discovery Grant No. 385773.
